# Therapeutic Agents with AHR Inhibiting and NRF2 Activating Activity for Managing Chloracne

**DOI:** 10.3390/antiox7070090

**Published:** 2018-07-13

**Authors:** Masutaka Furue, Yoko Fuyuno, Chikage Mitoma, Hiroshi Uchi, Gaku Tsuji

**Affiliations:** 1 Department of Dermatology, Kyushu University, Maidashi 3-1-1, Higashiku, Fukuoka 812-8582, Japan; y-kuba@dermatol.med.kyushu-u.ac.jp (Y.F.); mchikage@dermatol.med.kyushu-u.ac.jp (C.M.); uchihir@dermatol.med.kyushu-u.ac.jp (H.U.); gakku@dermatol.med.kyushu-u.ac.jp (G.T.); 2 Research and Clinical Center for Yusho and Dioxin, Kyushu University, Fukuoka 812-8582, Japan; 3 Division of Skin Surface Sensing, Kyushu University, Fukuoka 812-8582, Japan

**Keywords:** aryl hydrocarbon receptor, chloracne, dioxin, nuclear factor-erythroid 2-related factor-2, heme oxygenase-1, Yusho

## Abstract

Chloracne is the major skin symptom caused by dioxin intoxication. Dioxin activates the aryl hydrocarbon receptor (AHR)–cytochrome p450 1A1 (CYP1A1) system, generates oxidative stress, and induces hyperkeratinization of keratinocytes and sebocytes leading to chloracne. Nuclear factor-erythroid 2-related factor-2 (NRF2) is a master switch that induces the expression of various antioxidative enzymes, such as heme oxygenase-1. Cinnamaldehyde is an antioxidant phytochemical that inhibits AHR–CYP1A1 signaling and activates the NRF2–antioxidative axis. The cinnamaldehyde-containing Kampo herbal medicine *Keishibukuryogan* is capable of improving chloracne in Yusho patients who are highly contaminated with dioxin. Agents with dual functions in promoting AHR–CYP1A1 inhibition and NRF2 activation may be useful for managing dioxin-related health hazards.

## 1. Introduction

Health problems associated with environmental pollutants are an important issue. Environmental polycyclic aromatic hydrocarbons such as 2,3,7,8-tetrachlorodibenzo-*p*-dioxin, polychlorinated dibenzofuran, and benzo(a)pyrene (BaP) are high-affinity ligands for the aryl hydrocarbon receptor (AHR) or dioxin receptor [1,2,3,4]. These chemical compounds strongly activate AHR, generate reactive oxygen species (ROS), and induce the production of inflammatory cytokines in various tissues including skin [1,2,3,4]. To maintain cellular homeostasis, excessive production of ROS should be neutralized or minimized by cellular antioxidants, including antioxidative enzymes such as heme oxygenase-1 (HMOX1) and NAD(P)H:quinone oxidoreductase 1 (NQO1) [5,6]. The induction of these antioxidative enzymes is upregulated by nuclear factor-erythroid 2-related factor-2 (NRF2), which is a master transcription factor for antioxidant signaling [3,5,6].

Exposure to high concentrations of dioxin induces various acute and chronic health hazards including general fatigue, and neurological (numbness or pain in the limbs), respiratory (cough and sputa), and dermatological symptoms [7,8,9]. In addition, high-dose dioxin intoxication increases the prevalence of cardiovascular diseases, hyperlipidemia, thyroid diseases, diabetes, liver dysfunction, and chronic bronchitis [7,10,11]. Moreover, blood concentrations of dioxins are correlated with conditions, such as general fatigue, increased blood sugar, and hyperlipidemia [12]. The increased rate of mortality associated with liver and lung cancers is an additional important issue in dioxin intoxication [13,14]. 

Among the cutaneous symptoms caused by dioxin, chloracne is one of the major ones, causing significant deterioration in the quality of daily life [15,16,17,18]. Chloracne has a characteristic skin distribution, frequently affecting the retroauricular and malar areas of the face, ear lobes, and groin, whereas the nose and perioral area are typically spared [16,17,18,19,20]. The severity of chloracne is also correlated with the blood dioxin level [16]. 

The pathology of chloracne is characterized by hyperkeratinization of the interfollicular epidermis, hyperproliferation and hyperkeratinization of hair follicle cells, gradual loss of sebocytes with shrinkage of sebaceous glands, and infundibular dilatation, eventually leading to comedo formation [3,16,17,18,21,22]. AHR is abundantly expressed in epidermal keratinocytes and sebocytes [3,21]. Moreover, highly lipophilic dioxins appear to accumulate in, and are excreted via, sebaceous glands and sebum [19,23,24], which facilitates dioxin excretion from the intoxicated body [25]. The high concentration of dioxin in the sebum may explain why chloracne frequently develops in individuals with high-dose dioxin intoxication.

In accordance with the histopathology of chloracne, agonistic ligation of AHR accelerates epidermal terminal differentiation and keratinization [26,27,28]. Upon AHR stimulation, the proliferation and lipid synthesis of sebocytes are impaired, probably due to the switching of sebocytes toward keratinocyte-like differentiation [21,29,30]. In this review, we focus on the AHR signaling related to chloracne and highlight its potential treatment with an NRF2 agonist.

## 2. AHR Signaling in Keratinocytes and Sebocytes

**Figure 1:**
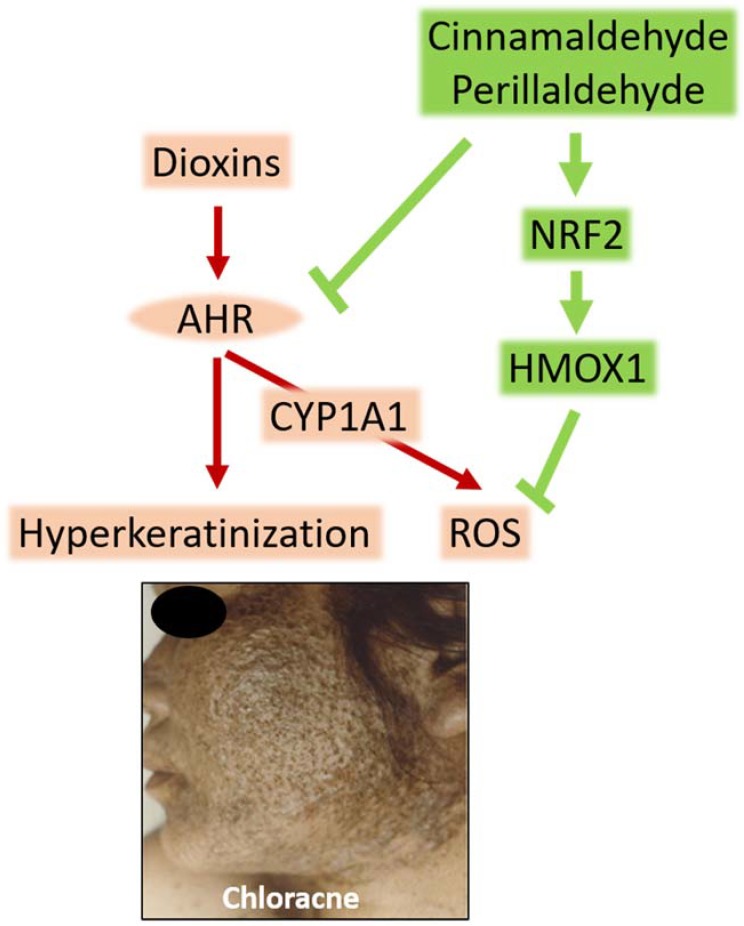
Dioxins activate the aryl hydrocarbon receptor (AHR), upregulate the expression of cytochrome P450 1A1 (CYP1A1), and generate reactive oxygen species (ROS) in keratinocytes and sebocytes. The ligation of AHR by dioxins also accelerates terminal differentiation. Oxidative stress and hyperkeratinization are probably responsible for chloracne. Cinnamaldehyde (a functional component of *C. cassia*) and perillaldehyde (a functional component of *P. frutescens*) are potent inhibitors of AHR–CYP1A1 signaling. On the other hand, they activate nuclear factor-erythroid 2-related factor-2 (NRF2). NRF2 is a master switch for the cellular antioxidative system. The activation of NRF2 upregulates various antioxidative enzymes, such as heme oxygenase-1 (HMOX1), and neutralizes ROS. These natural phytochemicals are useful for managing chloracne.

As a chemical sensor, AHR is constitutively expressed in the tissues, separating the inside and outside of the body, including the epidermis and pilosebaceous units [2,3,31]. Dioxins activate AHR and induce its cytoplasmic-to-nuclear translocation. Nuclear AHR binds to its specific DNA recognition site, namely, the xenobiotic responsive element and upregulates the transcription of responsive genes, such as cytochrome p450 1A1 (CYP1A1) in keratinocytes and sebocytes [2,3,31]. CYP1A1 is a xenobiotic-metabolizing enzyme and metabolizes dioxin [1]. As dioxin is very stable and persistent, the metabolizing process by CYP1A1 generates high levels of ROS (Figure 1). In CYP1A1-deficient conditions, ROS production is profoundly attenuated [4,32]. ROS-mediated oxidative stress induces DNA damage and upregulates the production of inflammatory cytokines and chemokines, such as IL-8 and CCL2 from keratinocytes [1,4,33].

In addition to generating oxidative stress, persistent activation of AHR by dioxin accelerates the terminal differentiation of keratinocytes and epidermal hyperkeratosis [27,28]. This effect is mediated by coordinated upregulation of the gene expression of epidermal terminal differentiation molecules, such as filaggrin and proline-rich small proteins (Figure 1) [26,34].

Upon AHR activation by dioxin, sebocytes lose their specific features for sebaceous differentiation, including lipogenesis, keratin 7 expression, and epithelial membrane antigen expression [21]. Instead, AHR ligation converts sebocytes towards keratinocytic differentiation, upregulating keratin 10 and peroxisome proliferator-activated receptor-δ [21]. These findings have been corroborated by ex vivo sebaceous gland cultures where it has been shown that dioxin induces the shrinkage and disappearance of sebaceous glands [21]. These keratinocytic and sebocytic alterations by dioxin coincide with the pathological features of chloracne [18,22].

## 3. Role of NRF2 in Neutralizing AHR-Mediated Oxidative Stress

Under unstimulated conditions, NRF2 resides in the cytoplasm, but upon activation, it translocates to the nucleus. The antioxidative enzymes downstream of NRF2 include HMOX1, NQO1, glutathione S-transferase, UDP-glucuronosyltransferases, epoxide hydrolase, glutathione reductase, thioredoxin reductase, catalase, and superoxide dismutase. NRF2 also activates the transcription of genes encoding non-enzymatic antioxidative proteins, such as thioredoxin and ferritin [6].

Dioxin induces AHR-mediated ROS production [26,35]. The oxidative stress reciprocally activates the NRF2–antioxidative pathway in order to neutralize excessive ROS generation [36]. However, dioxin is structurally stable and is very difficult to degrade. Therefore, dioxin is capable of activating AHR for a long period. Therefore, persistent activation of the AHR–oxidative pathway by chemically stable dioxin may overwhelm NRF2–antioxidative signaling, leaving the cell in a ROS-rich milieu.

A variety of salubrious, antioxidative plants and herbs utilize NRF2 to exert antioxidative activity. For example, phytoextracts from artichoke in Mediterranean countries, cactus *Opuntia ficus-indica* in Mexico, and the Asian herb, *Houttuynia cordata*, inhibit BaP/AHR-mediated oxidative stress via NRF2 activation [37,38,39]. Moreover, NRF2-mediated antioxidative activity is capable of alleviating ROS production induced by tumor necrosis factor-α [37,38,39]. These results highlight that exogenous NRF2 agonists can antagonize dioxin–AHR–ROS signaling.

## 4. Therapeutic Potential of *Cinnamomum cassia*-Containing Kampo Herbal Medicine for Chloracne

As antioxidant phytoextracts are potent inhibitors of AHR-mediated oxidative stress, we screened phytoextracts that inhibit the AHR–CYP1A1 pathway and activate the NRF2–antioxidative pathway. *Cinnamomum cassia* extract and its major constituent cinnamaldehyde have dual activity [40]. Both *C. cassia* extract and cinnamaldehyde attenuate the AHR–CYP1A1 axis and inhibit oxidative stress [40]. Many Japanese Kampo herbal medicines contain varying doses of *C. cassia* extract. Among them, *Keishibukuryogan* is the strongest inhibitor of AHR–CYP1A1 signaling [40]. In addition, both *C. cassia* extract and cinnamaldehyde activate the NRF2–HMOX1 antioxidative system and inhibit AHR-mediated ROS production (Figure 1) [40].

We conducted a clinical trial of the oral administration of *Keishibukuryogan* to treat Yusho patients who had been intoxicated with high concentrations of polychlorinated dibenzofurans after they ate a contaminated rice bran oil in 1968 [8,9]. Their mean blood concentrations of polychlorinated dibenzofurans still remained more than 10 times higher than that of normal individuals 30 and 40 years after the accident [41,42]. They suffer from chloracne, general fatigue, numbness and paresthesia of the extremities, cough, and expectoration of sputum [9]. After 3 months of oral administration, *Keishibukuryogan* significantly attenuated the symptoms of chloracne, general fatigue, and cough, and expectoration of sputum. It also tended to reduce symptoms of numbness and paresthesia of the extremities [9].

Perillaldehyde is another useful phytochemical [33]. It is a flavoring ingredient found in *Perilla frutescens* (*shiso* in Japanese), which adds spiciness and a citrus taste to food. Like cinnamaldehyde, perillaldehyde inhibits AHR–CYP1A1 signaling and activates the NRF2–HMOX1 antioxidative axis [33]. Unfortunately, *P. frutescens* extract-containing Kampo medicines do not exhibit similar activities, suggesting that dried *P. frutescens* extract may lose the flavoring of perillaldehyde during the extraction process. However, consuming fresh *P. frutescens* in meals on a daily basis may be helpful in managing chloracne.

## 5. Conclusions

Chloracne is a devastating skin symptom induced by exposure to high concentrations of dioxins and other hazardous compounds. These environmental pollutants bind to and activate AHR and generate abundant ROS. They also accelerate the terminal differentiation and keratinization of keratinocytes and sebocytes. As dioxin is stable and resistant to metabolization, persistent activation of AHR results in exaggerated oxidative stress and unopposed hyperkeratinization. These features probably explain the pathogenesis of chloracne.

Cinnamaldehyde and perillaldehyde are potent phytochemicals that inhibit the AHR–CYP1A1 pathway and activate the NRF2–antioxidative axis [33,40]. Given that cinnamaldehyde-containing herbal medicine improves the clinical symptoms of patients with dioxin intoxication, agents with dual functions in promoting AHR–CYP1A1 inhibition and NRF2 activation are potential candidates for managing dioxin hazards. Since *C. cassia* and *P. frutescens* are inexpensive and popular plants in Asia, their daily ingestion may be a suitable approach for defending against the health hazards of people living in areas contaminated with high levels of dioxins.
